# Reply to Rourk, C. Comment on “Albantakis et al. Computing the Integrated Information of a Quantum Mechanism. *Entropy* 2023, *25*, 449”

**DOI:** 10.3390/e25101442

**Published:** 2023-10-12

**Authors:** Larissa Albantakis, Robert Prentner, Ian Durham

**Affiliations:** 1 Department of Psychiatry, University of Wisconsin-Madison, Madison, WI 53719, USA; 2 Association for Mathematical Consciousness Science, 80539 Munich, Germany; robert.prentner@amcs.science (R.P.); idurham@anselm.edu (I.D.); 3 Munich Center for Mathematical Philosophy, Ludwig-Maximilians-University Munich, 80539 Munich, Germany; 4 Department of Physics, Saint Anselm College, Manchester, NH 03102, USA

**Keywords:** causal analysis, causation, quantum information theory

## Abstract

In response to a comment by Chris Rourk on our article *Computing the Integrated Information of a Quantum Mechanism*, we briefly (1) consider the role of potential hybrid/classical mechanisms from the perspective of integrated information theory (IIT), (2) discuss whether the (Q)IIT formalism needs to be extended to capture the hypothesized hybrid mechanism, and (3) clarify our motivation for developing a QIIT formalism and its scope of applicability.

In a comment to our original article *Computing the Integrated Information of a Quantum Mechanism* [1], the comment author raises the issue of hybrid quantum/classical mechanisms, which might underlie some biological computing systems, including the human brain [2]. Whether such hybrid mechanisms are indeed of relevance within the human substrate of consciousness is an interesting scientific question that we will remain agnostic about in the following. In general, the causal model to which (Q)IIT is eventually applied should adequately reflect all relevant physical details of the system under investigation. In other words, if hybrid mechanisms such as the hypothesized quantum adiabatic energy routing mechanism play a role in the proper functioning of the brain at the relevant spatio-temporal scale corresponding to our conscious experiences, they should be incorporated into the causal model to which the (Q)IIT formalism is applied.

The question is whether the (Q)IIT formalism needs to be extended to capture the hypothesized hybrid biological computing systems. As correctly stated in the comment, IIT/QIIT is formulated to be generally applicable to discrete/finite dimensional systems. The restriction to discrete units and updates in IIT is not only a matter of simplification but also reflects a commitment of IIT to the principles of “operational physicalism” and “operational reductionism” [3], which imply that it should be possible to account for everything that can be manipulated and observed purely in terms of cause–effect power. Nevertheless, in physics, but also in biology or neuroscience, processes are often described in continuous time and thus would have to be discretized for IIT’s formal framework to be applicable. With respect to consciousness, what matters according to IIT are the unit-, time-, and state-grains over which the system specifies a maximum of integrated information [3]. Notably, this can happen at coarser (more macroscopic) grains under certain conditions [4,5]. With respect to the hybrid mechanism proposed in the comment, its relevant variables (cortical inputs, neurons, and electrons) already seem to be discrete. It is thus not immediately clear to us that a continuous treatment is, in fact, necessary. For future work, we would encourage the construction of a simplified toy model to test both the functionality of the proposed hybrid mechanism and to what extent it could be discretized into a causal model as required by the IIT formalism.

Finally, we want to take this opportunity to clarify both our motivation for developing a quantum IIT formalism and its scope of applicability. As mentioned above, IIT postulates that the spatio-temporal grain of consciousness corresponds to a maximum of integrated information across all possible levels of description of a system. A comparison between quantum and classical systems is thus relevant to IIT because consciousness seems to be a macroscopic phenomenon that does not rely on the microscopic quantum processes that constitute neurons or other parts of the brain. In other words, the quantum processes to be compared are not different mechanisms from those of neural interactions but the same mechanisms described at a much more fine-grained quantum level. By contrast, the comment suggests a potential role for quantum processes at the macro level of description corresponding to consciousness. While the QIIT article evaluates quantum gates such as the CNOT as example systems to demonstrate certain features of the causal analysis, the QIIT formalism applies quite generally to finite dimensional “CPTP” (completely positive trace-preserving) maps, which include all possible finite dimensional unitary evolutions.
